# The Role of Transforming Growth Factor-β Signaling in Myxomatous Mitral Valve Degeneration

**DOI:** 10.3389/fcvm.2022.872288

**Published:** 2022-05-17

**Authors:** Qiyu Tang, Andrew J. McNair, Kanchan Phadwal, Vicky E. Macrae, Brendan M. Corcoran

**Affiliations:** ^1^The Roslin Institute, The University of Edinburgh, Edinburgh, United Kingdom; ^2^Royal (Dick) School of Veterinary Studies, The University of Edinburgh, Edinburgh, United Kingdom

**Keywords:** MVP, MMVD, valve interstitial cell, valve endothelial cell, TGF-β, BMP

## Abstract

Mitral valve prolapse (MVP) due to myxomatous degeneration is one of the most important chronic degenerative cardiovascular diseases in people and dogs. It is a common cause of heart failure leading to significant morbidity and mortality in both species. Human MVP is usually classified into primary or non-syndromic, including Barlow’s Disease (BD), fibro-elastic deficiency (FED) and Filamin-A mutation, and secondary or syndromic forms (typically familial), such as Marfan syndrome (MFS), Ehlers-Danlos syndrome, and Loeys–Dietz syndrome. Despite different etiologies the diseased valves share pathological features consistent with myxomatous degeneration. To reflect this common pathology the condition is often called myxomatous mitral valve degeneration (disease) (MMVD) and this term is universally used to describe the analogous condition in the dog. MMVD in both species is characterized by leaflet thickening and deformity, disorganized extracellular matrix, increased transformation of the quiescent valve interstitial cell (qVICs) to an activated state (aVICs), also known as activated myofibroblasts. Significant alterations in these cellular activities contribute to the initiation and progression of MMVD due to the increased expression of transforming growth factor-β (TGF-β) superfamily cytokines and the dysregulation of the TGF-β signaling pathways. Further understanding the molecular mechanisms of MMVD is needed to identify pharmacological manipulation strategies of the signaling pathway that might regulate VIC differentiation and so control the disease onset and development. This review briefly summarizes current understanding of the histopathology, cellular activities, molecular mechanisms and pathogenesis of MMVD in dogs and humans, and in more detail reviews the evidence for the role of TGF-β.

## Introduction

Mitral valve prolapse (MVP) is one of the most common cardiac valvular abnormality in dogs and humans, and is a major source of morbidity and mortality and a common cause of heart failure, ventricular dysfunction, arrhythmias and sudden cardiac death (1–6). In the dog the condition is more commonly called myxomatous mitral valve disease (MMVD). Since this mitral valvulopathy in both species has various synonyms and myxomatous changes is the predominant pathological finding, in this review the terms MVP and MMVD will be used interchangeably and when needed for human and dog, respectively. MVP affects to 2–3% of the human population, and more than 10% of individuals over the age of 65 years have mitral valve insufficiency (7, 8). It accounts for 7% of deaths in dogs before 10 years of age and its prevalence is very high, estimated between 30 and 70% of all elderly dogs, with the greater prevalence in small breed dogs and in certain predisposed breeds such as the cavalier King Charles spaniel (CKCS) (9–11). Some forms of MVP will have a congenital or genetic basis, meaning MVP can be further characterized into primary non-syndromic, and secondary syndromic forms (4). Syndromic MVP is commonly associated with global genetic connective tissue disorders, such as Marfan syndrome (MFS), Loeys-Dietz syndrome and Ehlers-Danlos syndrome (12–14). It can also appear as isolated non-syndromic MVP, typically in a familial setting. The first confirmed non-syndromic genetic mutation was for the X-linked FLNA (Filamin-A) gene mutation, which causes valvular defects and progressive myxomatous degeneration and MVP in mice and humans (15–17). For the non-syndromic variants two disease types are reported including Barlow’s Disease (BD), where there is myxomatous degeneration and end-stage fibrosis, and fibroelastic deficiency (FED) where there is only myxomatous degeneration with valve thinning rather than thickening (18). FED is typically only seen in the very elderly, while Barlow’s Disease (BD) has more commonality with canine MMVD in terms of its slowly acquired development and progression, despite the lack of fibrosis in the dog. Lastly, MMVD has also been described in mice and pigs, induced by gene interference or surgical intervention, but the extent of MMVD in other species as a consequence of aging is unknown (19–21). Details of MMVD in both species are summarized in Table 1.

**TABLE 1:** Characteristics of various forms of MMVD in the human and dogs.

Classification			Histopathological features	Abnormal signaling	Cellular changes
Human MVP	Primary (Non-syndromic)	Barlow’s Disease (BD)	1. Typical lesions of myxomatous degeneration 2. End-stage fibrosis 3. Leaflet thickening	1. TGF-β signaling 2. 5-HT (serotonin) signaling 3. BMP signaling 4.Wnt/β-catenin	1. Increased aVICs from qVICs 2. Increased macrophage infiltration 3. EndoMT
		Fibroelastic deficiency (FED)	1. Typical lesions of myxomatous degeneration 2. Deficiency in collagen 3. Valve thinning	TGF-β signaling	Increased aVICs from qVICs
		Filamin-A mutation	Typical lesions of myxomatous degeneration	1. TGF-β signaling, 2. Ras/Mek/Erk signaling 3. 5-HT (serotonin) signaling	1. Increased aVICs from qVICs
	Secondary (Syndromic forms)	Marfan syndrome (MFS)	Typical lesions of myxomatous degeneration	1. TGF-β signaling 2. BMP signaling 3. Wnt/β-catenin	1. Increased aVICs from qVICs 2. Increased macrophage infiltration 3. EndoMT
		Ehlers-Danlos syndrome	Typical lesions of myxomatous degeneration		
		Loeys–Dietz syndrome	Typical lesions of myxomatous degeneration	TGF-β signaling	
Canine MMVD			1. Typical lesions of myxomatous degeneration 2. Lack of any fibrotic changes	1. TGF-β signaling 2. 5-HT (serotonin) signaling 3. BMP signaling	1. Increased aVICs from qVICs 2. EndoMT 3. Lack of inflammatory infiltration

For both syndromic and non-syndromic forms in both species the diseased valves appear histologically similar and exhibit features of myxomatous (myxoid) degeneration. Progressive deterioration of the mitral valves is typically characterized by increased valvular nodularity, leaflet thickening and deformity, excessive accumulation of proteoglycans (GAGs), collagen and elastin fragmentation, increased expression of proteolytic enzymes, disorganized extracellular matrix (ECM) and increased numbers of activated valve interstitial cells (aVICs; activated myofibroblasts) and with more obvious macrophage infiltration in human valves (3, 5, 22–25). Furthermore, in human valves there can be additional fibrosis in end-stage disease (BD) characterized by fibrotic layers on the valve surface, a change not seen in the dog (26–29).

Although, the histopathological changes of myxomatous degeneration are well characterized, some aspects of the underlying molecular changes and its contribution to pathogenesis are still to be identified (22, 30, 31). Most MMVD seen in human and veterinary clinical practice are sporadic and of unknown etiology, although a genetic or inherited basis suspected. However, there is increasing evidence for a primary role for members of the transforming growth factor (TGF)-β superfamily in the pathogenesis and progression of various MMVD forms (5, 32–37). This is not surprising as the TGF-β superfamily are important in the regulation of most cellular events, such as proliferation, differentiation, migration, autophagy, apoptosis and senescence in a variety of cardiovascular diseases (38, 39). While much is known about the cellular and molecular events of the disease in both species, the exact molecular and regulatory mechanisms are not yet elaborated, especially with regard to early disease onset and progression (3–5, 37). In human MVP most studies of non-syndromic forms have been restricted to examining end-stage valves obtained at surgery (29, 34, 35, 40). Information on earlier onset is restricted mainly to inherited connective tissue disorders, such as MFS, osteogenesis imperfecta, and Ehlers-Danlos syndrome, although myxomatous degeneration in those patients can appear with advanced age (17).

CD45^+^ hematopoietic cells have been detected in human, sheep, and murine MMVD valves related to non-infective causes, although MMVD in the absence of infective endocarditis has traditionally been regarded as a non-inflammatory disease (34, 41–44). The majority of these cells are characterized as macrophages implicating macrophage infiltration as a potential secondary driver of MMVD progression. CD45 expression of mitral VECs can be induced by TGF-β signaling in sheep MMVD (25, 43, 45). TGF-β signaling is enhanced in MMVD in mouse, humans and dogs and has been associated with ECM dysregulation and increased macrophage numbers in diseased valves (4, 32, 34, 46, 47). These data suggest that TGF-β signaling might be associated with the emergence of an inflammatory micro-environment comprised of increased recruitment of pro-inflammatory macrophages from the circulation and immunogenic ECM remodeling, somewhat analogous to features of calcific aortic valve disease (CAVD) (6, 25). In CAVD TGF-β signaling is involved in the pathogenesis, as for MMVD a promoter role in the early VIC activation as shown by up-regulation of α-smooth muscle actin (α-SMA). In dogs with advanced disease there is evidence of a marginal increase in the number of mast cells in affected valves (24), but no involvement of the resident macrophage population or recruitment of inflammatory cells are found in severe MMVD (elderly dogs) (48, 49). Whether there is a macrophage population present in dogs or only during early stage of the disease has yet to be elucidated.

## Valve Structure, Pathology, and Cell and Molecular Changes

**FIGURE 1:**
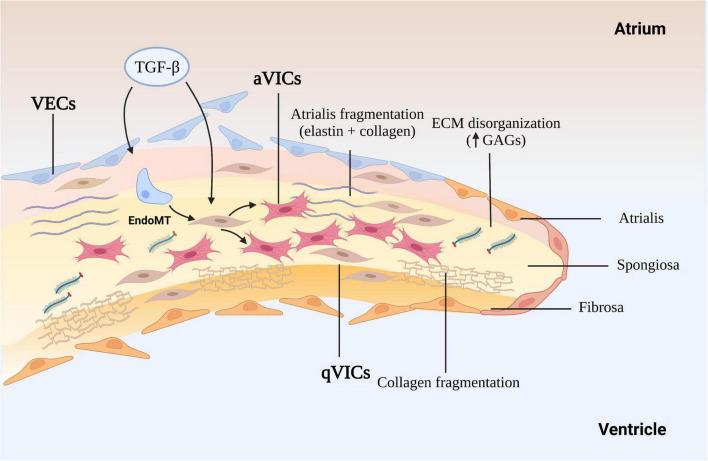
The mechanism of myxomatous degeneration. The schematic shows the endothelial to mesenchymal transition (EndoMT) process and the activation of quiescent valve interstitial cells to myofibroblasts that affect matrix production and remodeling, driving collagen and elastin fragmentation and loss. ECM, extracellular matrix; GAGs, glycosaminoglycans; MMP, matrix metalloprotease; TGF-β, transforming growth factor-β; qVICs, quiescent valvular interstitial cells; aVICs, activated valvular interstitial cells.

The cross-sectional structure of the normal mitral leaflets is similar to the aortic valve, with at least three layers, atrialis, spongiosa, and fibrosa identified, a fourth ventricularis proposed by some, and each with different thickness and cell and ECM composition, and both sides lined with valve endothelial cells (VECs). The thin atrialis contains a large amount of elastin with a mixed amount of scattered collagen fibers and valve interstitial cells (VICs) (50, 51). The spongiosa contains loosely arranged collagen fibers and is rich in glycosaminoglycans (GAGs), such as hyaluronan, and various proteoglycans (52). This layer, consisting mainly of collagen I and III and small numbers of thin elastin fibers. The majority of cells in the spongiosa, and throughout the whole valve, are quiescent VICs (qVICs) along with few mast cells (24, 53–55). The dense fibrosa layer is composed of tightly packed collagen bundles arranged parallel to the leaflet free edge and within which are scattered VICs (Figure 1).

The gross and histopathological changes in both species are reasonably well characterized, and will be only briefly describe here, but to give contextual background to the role of TGF-β in the disease process (5, 22, 37, 56–58). Changes the endothelium include endothelial loss, cellular pleomorphism, endothelial-to-mesenchymal transition (EndoMT), disruption of the basement membrane and accumulation of aVICs in the sub-endothelium (23, 46, 55). The myxomatous degeneration itself is characterized by expansion of the spongiosa and separation as well as fragmentation of the dense collagen bundles in the fibrosa (5, 59). There is a reduction in connective tissue density, accumulation of a myxoid extracellular matrix rich in GAGs, loss of mature collagen and replacement with immature fibrillar collagen lacking cross-links structural, in all the layers of leaflet as well as the chordae tendineae (60). The main cellular event is the phenotypic differentiation of the VICs from a quiescent phenotype (qVICs) to an activated myofibroblast (aVICs) in the spongiosa and fibrosa, with accumulation of aVICs in the sub-endothelium (46, 61). Furthermore, there is evidence of aVIC persistence with dysregulation of apoptosis, and transcriptomic evidence of altered gene expression associated with cellular senescence (37, 62). Rather than aVICs being cleared for the tissue as would normally happen, their persistence might contribute to the aberrant matric remodeling typical of the disease. The aVICs are presumed responsible for valve matrix degrading, and at a rate exceeding that of production of new collagen and elastin (63). This matrix degeneration is presumed to be due to increased production of various proteolytic enzymes including matrix metalloproteinases (MMP-1, MMP-2, MMP-9, and MMP-13). It should be noted that human valvulopathies heighten the risk of developing endocarditis which is not the case in the dog. The reason for this species difference is unknown. Overall, these pathological changes account for the reduced tensile strength, distorted valve shape and mechanics typical of the disease resulting in the mitral regurgitation and heart failure.

In addition to the naturally occurring forms of MMVD there are various rodent models available, and comment will be made on how they inform thinking on the role of TGF-β (Table 2). Many of these models are transgenic and questions can be raised to their validity as models of what is a chronic degenerative disease, but that discussion is beyond the scope of this review.

**TABLE 2:** *In vivo* and *in vitro* models for MMVD.

Classification	*In vivo* animal models	*In vitro* culture models
Myxomatous mitral valve degeneration (MMVD)	1.Mouse treated with AngII (84) 2.Mouse treated with nordexfenfluramine (NDF) (133, 134) 3. FVB mouse (135)	1.Porcine 3D mitral VIC culture system (136) 2.Porcine mitral VIC culture system (100) 3.Human mitral valve tissue culture system (34) 4.Human mitral VIC culture system (34, 114)
Barlow’s Disease (BD)	Dogs with spontaneous MMVD (5, 37)	1.Canine 2D mitral VIC and VEC co-culture system (36) 2.Canine 3D tissue-engineered fibrin-based cell culture system (137)
Filamin-A mutation	Filamin-A KO mouse (89)	
Marfan syndrome (MFS) (32, 107)	Fbn1^C1039G/+^ mouse Fbn1*^C1039G/C1039G^* mouse	
Ehlers-Danlos syndrome (138)	Col3a1± mouse Col5a1± mouse	
Loeys–Dietz syndrome (117)	TGF-βR1± mouse TGF-βR2± mouse TGF-βR1^M318R/+^ mouse TGF-βR2^G357W/+^ mouse	

## The Role of Transforming Growth Factor-β in Myxomatous Mitral Valve Degeneration

### Effects of Transforming Growth Factor-β on Valvular Interstitial Cells

As mentioned previously, VICs exist in all layers as two distinct phenotypes, qVICs and aVICs, and predominantly in healthy and diseased valves, respectively (64). During development endothelial cells invade the endocardial cushion and are transformed into embryonic progenitor mesenchymal cells to induce ECM remodeling under the regulation of the TGF-β family and bone morphogenetic proteins (BMPs) (65). Once the valve is formed qVICs predominate and maintain valve structure and function (24, 48, 54). VICs in normal valves have a quiescent vimentin^+^/alpha-smooth muscle actin (αSMA)- phenotype and are presumed to operate in a homeostatic role controlling ECM remodeling and repair (66). TGF-β up-regulation appears to have an important role among various biological pathways in the pathogenesis of the multiple forms of MMVD. Specifically, TGF-β is known to activate qVICs toward a pathologic synthetic phenotype, as shown both in animal models and in human and canine *in vitro* studies (32, 36, 48). Similarly, antagonism of the TGF-βR II receptor by SB431542 transitions aVICs to the qVIC phenotype in a canine low serum culture system, where VICs were isolated from diseased canine mitral valves and maintained in 2% (v/v) FBS media (36). Members of the TGF-β superfamily are overexpressed in surgically excised human diseased valves where aVICs predominate and this is associated with increased expression of MMPs, presumably driving degeneration of collagen and elastin structures (22, 34, 35, 67, 68). Finally, examining transcriptomic data from human valves, upregulation of BMP 4 has been shown to mediate the activation of VICs from healthy quiescent cells to a pathologic synthetic phenotype (67).

### Transforming Growth Factor-β Control of EndoMT in Myxomatous Mitral Valve Degeneration

**FIGURE 2:**
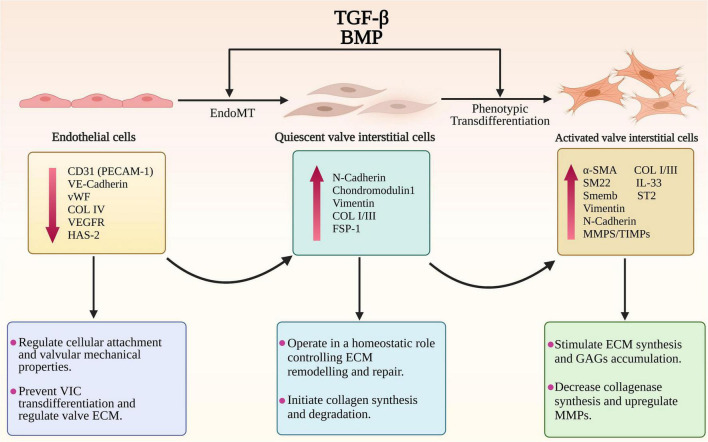
The endothelial to mesenchymal transition (EndoMT) and phenotypic trans-differentiation from quiescent valve interstitial cells (qVICs) to activated valve interstitial cells (aVICs) in the progression of MMVD. The diagram illustrates the phenotypic, gene expression changes and cellular functional changes occurring during MMVD. The phenotypic conversion of endothelial cells into qVICs includes increased production of N-cadherin, vimentin and fibroblast-specific protein-1 (FSP-1). These events are accompanied by downregulation of the markers of endothelial cells such as CD31/platelet-endothelial cell adhesion molecule-1 (CD31/PECAM-1), vascular-endothelial cadherin (VE-cadherin), COL4, vascular epidermal growth factor receptor (VEGFR), and von Willebrand factor (vWF). Furthermore, the TGF-β-activated VICs show a significant increase in unique markers including α-smooth muscle actin (α-SMA), SM22 and Smemb (embryonic smooth muscle myosin) as well as the secretory proteins regulating the ECM disorganization including MMPs/TIMPs, IL-1β, IL-11, and IL-33.

One of the important, but less documented, changes to the endothelium in MMVD is induction of EndoMT, accompanied by activation of transcriptional regulatory mechanisms important in heart valve development, and is seen in both human and canine MMVD (46, 69–71). This is associated with co-expression of hyaluronic acid synthase (HAS)-2 and α-SMA in endothelial cells and increased expression of Sox9, and increased expression of HAS-2 in stromal interstitial cells (46, 52, 72). There are other lines of evidence suggesting a role for EndoMT in MMVD, with a role for TGF-β. Cultured endocardial cells derived from mature ovine valves have been shown to transdifferentiate *via* TGF-β signaling into mesenchymal cells that express α-SMA, and human valve endothelial cells adopt a mesenchymal phenotype after exposure to TGF-β (73, 74). Clonal expansion has shown endothelial-like cells have a strong response to TGF-β, which can then be inhibited by vascular endothelial growth factor (VEGF) (74). *In vitro* mitral valve endothelial cells have been shown to be important for maintaining the quiescence of valve interstitial cells and thereby reciprocally preventing TGF-β-driven EndoMT of endothelial cells (75). Lastly, inhibition of TGF-β by the angiotensin two receptor antagonist losartan can reduce EndoMT of mitral valve endothelial cells *in vitro* in a sheep model and can decrease leaflet thickness and block TGF-β signaling and downstream targets (43). Nevertheless, evidence for EndoMT is restricted to valve transcriptomic data in the dog and human, and found in valve tissue in the dog using IHC, but to what extent this might contribute to the valve VIC population and to disease pathogenesis *in vivo* is unknown (Figure 2).

## Transforming Growth Factor-β Initiated Signaling Pathways in Myxomatous Mitral Valve Degeneration

The TGF-β family consists of a large variety of pleiotropic multifunctional proteins that play significant roles in embryonic development, autoimmunity, cancer, fibrotic disorders, and cardiovascular diseases (34, 76–79). TGF-βs and BMPs are considered as the most important initiators of the signaling pathway (80–83). Although the effect of TGF-βs and BMPs on the initiation and progression of MMVD has been shown, the molecular and regulatory events involved are highly complex resulting from a large number of signaling interactions promoted by diverse molecular mechanisms. Many of these interaction still need to be elucidated.

TGF-β-mediated valvular fibrosis is only observed in end-stage human MMVD and seen as fibrotic over-lays developing on the atrial and ventricular sides of leaflets. However the myxomatous degeneration found in both species is believed to be highly TGF-β-dependent (5, 37). Various studies have suggested the important roles for the members of TGF-β family in the initiation and development of MMVD in humans, mouse and dog (32–37, 47, 48, 67). Pathway analysis by transcriptomic profiling in human and canine valve tissue has identified TGF-β signaling as the dominant pathway in both the development and progression of MMVD (37, 84). Increased expression of multiple TGF-β isoforms in parallel with the accumulation of ECM components and transformation of VICs into myofibroblasts is observed in the surgically excised samples of myxomatous valves from human and dog (34, 37, 47, 85, 86). In a transgenic fibrillin (Fbn)-1-deficient mouse Marfan Disease model, where TGF-β signaling was potentiated, VICs are phenotypical altered with associated myxomatous ECM remodeling (32, 87). Transformation of qVICs to the diseased activated myofibroblast phenotype can be blocked with TGF-β-neutralizing antibodies (NeuAb), antagonism of the TGF-β RII receptor, antagonizing TGF-β signaling and blocking Smad phosphorylation (32, 34–36). In an *in vitro* Marfan Disease model an exon encoded Fbn-1 sequence triggers release of endogenous TGF-β1 and stimulates TGF-β receptor-mediated Smad2 signaling in the presence of cell layer ECM (88). However, it has been shown that non-Smad (non-canonical) signaling pathways are also implicated in MMVD progression, including regulation by several molecular mediators such as filamin A (FLN-A) and scleraxis (Scx) (86, 89).

### Canonical Transforming Growth Factor-β-Mediated Signaling Pathways in Myxomatous Mitral Valve Degeneration

**FIGURE 3:**
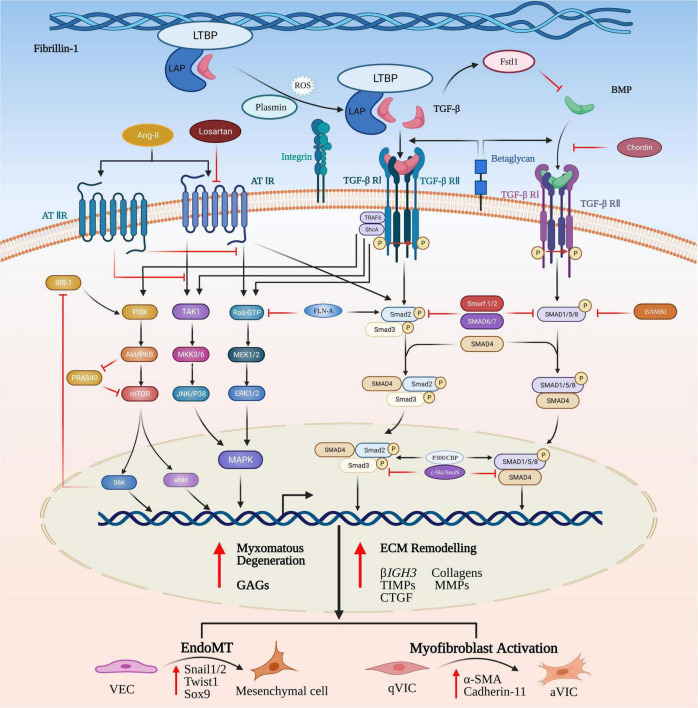
Canonical and non-canonical TGF-β and BMP signaling pathways in MMVD. TGF-β homodimers are secreted extracellularly as inactive protein complexes, maintaining latency through binding with the latency-associated peptide (LAP). Latent TGF-β-binding proteins (LTBPs) form a bridge between fibrillin-1 (Fbn-1) and LAP and serve to sequester the complex into the extracellular matrix (ECM). The complex can be released from the microfibrils by its complex interactions with activators such as reactive oxygen species (ROS), plasmin and integrins. Activation of the latent complex mechanically triggers the complex deformation (torqueing) and the release of active TGF-β. The Smad-mediated TGF-β cascade is initiated by the binding of activated TGF-β homodimers to their corresponding transmembrane heterodimeric TGF-β I/II receptor complex. The active receptor complex recruits and phosphorylates the downstream signaling effector proteins, Smad2 and Smad3. Upon phosphorylation, Smad2/3 is released and forms an intracellular complex with Smad4 that translocates from the cytoplasm into the nucleus, where it interacts with Smad binding elements of TGF-β target genes involved in induction of ECM remodeling, VIC differentiation, EndoMT and myxomatous alterations. The non-canonical pathways are comprised of phosphatidylinositol 3-kinase (PI3K) and the kinases members from mitogen-activated protein kinase (MAPK) family. TGF-β can activate all three known MAPK pathways, referred to as TGF-β activated kinase 1 (TAK1)-mediated p38 activation, c-Jun amino-terminal kinase (JNK) MAPK, and extracellular signal-regulated kinase (ERK). Signal transduction through these pathways modulates non-Smad-mediated TGF-β-responsive cellular activities dependent on a specific cellular type or context. In a similar manner, BMPs bind to their transmembrane complexes to initiate its phosphorylation, but with higher affinity with type I receptor, assisted by type III co-receptors, endoglin and betaglycan. Upon activation the complex results in initiation of canonical or non-canonical signaling cascades.

Substitution of an epidermal growth factor-like domain in the fibrillin-1 (Fbn-1) gene with a cysteine (C1039G) in transgenic mice will result in increased release of activated TGF-βs to initiate the aberrant signaling that contributes to myxomatous degeneration (32). Significant increased expression of TGF-βs, latency-associated peptides (LAPs), latent TGF-β activator integrins and phosphorylated SMAD2/3 has been reported in MMVD valve tissues using transcriptomic analysis and on histopathology, indicating the key contribution of canonical TGF-β-mediated signaling to MMVD (34, 37, 90, 91). Understanding how TGF-β signaling can control MMVD pathogenesis requires some explanation of the complex pathways involved. Briefly, the TGF-β signaling pathway is initiated by binding of activated TGF-β ligands with TGF-β receptor complexes in the cell membrane. Subsequent to intracellular biosynthesis TGF-β homodimers are secreted extracellularly as inactive protein complexes, which maintain latency through the non-covalent binding with the pro-peptide latency-associated peptide (LAP). To exert its diverse biological functions TGF-β requires to be liberated from the latent complex and activated extracellularly before binding to its receptors. For example, Fbn-1, as a structural component of the ECM microfibrils, can release (activate) latent TGF-βs from the microfibrils by means of the substitution of Fbn-1 fragments for latent TGF-β-binding proteins (LTBPs) and by its complex interactions with various activators such as integrins (37, 88, 92–95). TGF-β release can also be controlled by torqueing mechanics where the bound protein complex is linked to cells (in this case VICs) by various integrins. Following the activation/release of TGF-βs these homodimers bind to their corresponding transmembrane receptor complexes (TGF-βR I and TGF-βR II) and initiate a complicated intracellular signaling cascade of molecular and regulatory events (96–98). The active receptor complex recruits and activates the downstream signaling effector proteins Smad2 and Smad3 (the canonical pathway) and they form an intracellular complex with co-Smad (Smad4, the mediator Smad) enabling its translocation from the cytoplasm into the nucleus. In the nucleus the Smad complex interacts with the Smad binding elements (SBE) to invoke transcription of various TGF-β-responsive genes, including those involved in ECM remodeling, VIC differentiation and EndoMT (99) (Figure 3).

### The Role of Mediators in Canonical Transforming Growth Factor-β Initiated Myxomatous Mitral Valve Degeneration

While Smad2/3-mediated signaling probably is a major contributor to the initiating of MMVD, other molecular mediators related to canonical signaling pathways have been shown to play a significant role in the modulation of MMVD progression in animal models *in vivo* and *in vitro* in VICs. These regulatory mechanisms include molecules exerting their effects on MMVD by means of canonical TGF-β signaling pathways or through activation by TGF-β itself. Examples include fibrillin-1 (Fbn-1), filamin A (FLN-A), follistatin-like 1 (Fstl1), fibroblast growth factor (FGF)-2 and angiotensin (Ang) II (32, 84, 89, 100, 101).

Fbn-1 beyond being a structural component of ECM microfibrils has an important role in regulating TGF-β activity and signaling through interaction with LTBP in the myxomatous mitral valve of MFS patients (32). Ng and colleagues showed that the intense immunohistochemical signal of both active TGF-β and its intracellular responder pSmad2 were significantly increased in mitral valves in a MFS mouse model. These observations were further validated by TGF-β antagonism by neutralizing antibodies *in vivo* with rescuing of the myxomatous mitral valve phenotype. Additionally, the expression of the downstream TGF-β-related effectors, which are either members of the TGF-β superfamily or directly mediated by TGF-βs, such as βIGH3, endothelin-1 (EDN1) and BMPs, are upregulated in the mitral valves of MFS mice. This suggests a promoter role of the mutation in Fbn-1 in initiation of canonical TGF-β signaling in MFS, and perhaps more commonly non-syndromic variants of human MMVD (32). Evidence for the extracellular control by Fbn-1 of TGF-β signaling activation in other MFS-induced systemic disorders has been shown in other studies (102–108). Recent work on the age-dependent cardiac remodeling in a mouse model of MFS revealed a comparable expression pattern of the myofibroblast marker α-smooth muscle actin (α-SMA) in cardiac tissues of Fbn-1 transgenic mice and their wild-type littermates.

FLN-A is highly expressed in the mitral valve during development and is significantly diminished after birth, suggesting an important role for FLN-A during valve development (109). Mutations in the FLNA gene had been identified as a cause to a rare X-linked myxomatous valvular disease (17). FLN-A might act as a contributor to the initiation and development of myxomatous cardiac valves by its interaction with activated Smads to regulate TGF-β signaling (110, 111). In contrast, a recent study showed that FLN-A might not be involved in the pathogenesis of non-syndromic MVP, since the mRNA expression level of FLN-A in diseased valves is comparable to control leaflets, despite upregulation of TGF-β1 and pSmad2 signaling in diseased mitral valves (86). Another study also suggested that there were interactions between FLN-A and TGF-β since FLN-A acted as a promoter of TGF-β induced ECM remodeling in Fstl1-deficient mice (101). In a FLN-A knockout mouse the expression of the canonical TGF-β-dependent effector pSmad3 and its downstream target molecule collagen IαI are markedly increased in mitral valves suggesting deficiency in FLN-A positively influences Smad activation and correlates with increased collagen expression (89).

In Fstl1-knockout mice there is a sustained increase in TGF-β signaling after birth, while deletion of Fstl1 from the endocardial lineage results in myxomatous mitral valves with cell proliferation and endocardial-to-mesenchymal transition. Fstl1-deficient mitral valves show significantly upregulated expression of TGF-β in mitral valve VICs. Immunofluorescent analysis has shown that the positive signal for pSmad2/3 is mainly detected in the nuclei of VECs and VICs in diseased mitral leaflets, indicating active TGF-β signaling during postnatal development (101). Additional evidence that Fstl1 could regulate MMVD is shown by increased upregulation of a series of unique makers of ECM remodeling and EndoMT mediated by TGF-β signaling in Fst1-KO mice, including Fbn-1, FLN-A, vimentin and α-SMA. These data suggest important roles of Fstl1 in promoting homeostasis of the mitral valve undergoing embryonic development, postnatal maturation, and even into adulthood (101).

Several studies have shown that fibroblast growth factor (FGF)-2 is able to potentiate canonical pSmad2/3-dependent TGF-β signaling by binding to TGF-β type III receptor beta-glycan, which both possess the binding site of endogenous TGF-β and FGF-2, and thereby result in increased TGF-β availability for the activation of the canonical pathway. A significant role for FGF-2/AKT-1 signaling in mitral VICs in response to experimental wounding and remodeling, independent of the TGF-β/Smad signaling, has been identified (100). Ang II has also been shown to play a significant role in canonical TGF-β-dependent ECM remodeling causing myxomatous degeneration in murine mitral valves. Infusion of Ang II into mice triggers activation of a canonical Smad-mediated TGF-β2 cascade and transcriptional activity of TGF-β-responsive genes (84). There is increased mRNA levels of TGF-β1 and TGF-β2 as well as downstream pSmad2, detected by both qRT-PCR and IHC, alongside an increase in MMP2 and CTGF expression, respectively (84). As previously mentioned canonical TGF-β-induced SMAD2/3-dependent ECM production in cultured human VICs can be effectively inhibited by the Ang II receptor blocker losartan (34).

### Non-canonical Transforming Growth Factor-β Signaling in Myxomatous Mitral Valve Degeneration

In addition to the canonical signaling, TGF-β family members contribute to MMVD progression by *via* several non-Smad/non-canonical pathways. Similar to the Smad-dependent signaling, the non-canonical cascades are initially amplified by the phosphorylation of TGF-βRI/II through binding with activated TGF-β ligands regulating downstream cellular activities. These pathways comprise the kinases members from the mitogen-activated protein kinase (MAPK) family and other kinases such as phosphatidylinositol 3-kinase (PI3K). TGF-β can activate all three known MAPK pathways, and these are also known as TGF-β activated kinase 1 (TAK1)-mediated p38 activation, c-Jun amino-terminal kinase (JNK) MAPK, and extracellular signal-regulated kinase (ERK). Signal transduction through these pathways modulates non-Smad-mediated TGF-β-responsive cellular activities. However, these pathways can also modify canonical pathways with complex interactions between non-Smad- or Smad-mediated components occurring in most TGF-β-mediated biological effects. This can result in up- or down-regulation of TGF-β signaling and complex regulation of biological responses (112).

Although Smad-independent TGF-β signaling is implicated in a wide spectrum of intracellular transduction cascades and diverse cellular responses only a few studies have examined its involvement in MMVD (34, 41, 89, 113, 114). Nevertheless, these studies show that non-Smad signaling, including ERK, p38 MAPK, and PI3K, can contribute to MMVD pathogenesis affecting the function of both VICs and VECs by interacting with canonical TGF-β signaling or directly. Recent transcriptomic profiling of canine valve tissues has shown that positively regulated gene expression of the ERK1/2 cascade is most noticeably in end-stage valves (37). In FLNA conditional knockout mice, as found in the human FLNA mutation X-linked MMVD, progression can be attributed to a balance between the opposing regulatory effect of the non-canonical Ras/Mek/Erk and canonical TGF-β-dependent effector pSmad3 (89). In human mitral VICs exposed to cyclic mechanical strain, expression of TGF-β2 and α-SMA are significantly increased, which is partly dependent on the activation of RhoC/ROCK in tandem with the non-canonical MEK/ERK1/2 pathway (114). The expression of α-SMA induced by activation of the TGF-β-dependent PI3K/AKT signaling is also observed in bone marrow-derived mesenchymal stem cells (MSCs) modified to generate VICs for tissue-engineered heart valves (113). In human mitral VECs EndoMT can be induced *via* TGF-β-mediated activation of Ras/Mek/Erk rather than the canonical pSmad3 signaling with increased expression of the EndoMT markers Slug, Snai1 and MMP-2 (41). TGF-β-mediated ECM production has also been shown to be dependent on non-Smad p38 MAPK pathways, working in combination with the canonical Smad2/3 signaling in diseased VICs in sporadic non-syndromic mitral valve prolapse (34).

The potential interaction between Ang II and TGF-β in the pathogenesis of MMVD should also be considered as this is well recognized in fibrosis and ECM deposition, involving both canonical and non-canonical parts of the TGF-β signaling pathway, and these effects can be inhibited by angiotensin II receptor blockers (ARBs) (34). One consideration, as previously mentioned, is the disparity in the level of fibrosis comparing end-stage canine and human MMVD (BD), and to what extent signaling pathways more important in fibrosis might actually contribute to the ECM remodeling seen with myxomatous degeneration. Nevertheless, activation of Smad-mediated signaling and TGF-β2-responsive gene expression has been observed in the mitral valves of mice treated with Ang II, with a relatively moderate change in the activity of BMP and Wnt-β-catenin signaling, both of which are suggested to contribute to human MMVD (84). The ARB antagonist losartan competitively antagonizes binding of Ang II to the AT1 receptor, but also inhibits progression of aortic root aneurysms in Fbn-1 mutant mice and Marfan disease patients through anti-TGF-β effects (104, 115). It has been proposed that losartan shunts Ang II signaling toward the Type II receptor involving both SMAD and ERK (116). In human mitral VICs treated with losartan, phosphorylation of SMAD2/3 and p38 is inhibited, but not ERK, suggesting SMAD2/3-dependent canonical TGF-β and ERK-independent signaling contribute to MMVD progression. Furthermore, in a mouse model of Loeys-Dietz syndrome (LDS), which exhibits phenotypic features overlap with MMVD and is a consequence of dysregulated TGF-β signaling, losartan normalizes growth of aortic roots and protects the aortic wall from the damage associated with decreased expression of pSmad2, pERK and TGF-β1 ligands (117). Losartan antagonism of TGF-β-dependent pSmad and ERK signaling also prevents EndoMT in ovine VECs and reduces expression of TGF-β and pERK in sheep mitral valve tissues (43, 118). In transgenic Runx2± mice treated with AngII there is increased expression levels for the COL3A1 gene and immunostaining for pAKT proteins despite the lack of expression of TGF-β3 and pSMAD2/3 in mitral valve tissues. Conversely, there is increased expression of TGF-β3, COL3A1, p-SMAD2/3 and p-AKT in Runx2^+/+^ mice, suggesting that Runx2 may contribute to trigger tissue ECM responses and cellular proliferation, similar to that seen in MMVD (84). However, a large-scale clinical trial for children and young adults with MFS showed that there were no significant difference in the rate of aortic-root dilatation between the two groups treated with losartan and atenolol, the current standard therapy in most hospitals (119).

### Bone Morphogenetic Proteins Signaling in Myxomatous Mitral Valve Degeneration

BMPs are important members of the TGF-β superfamily and may also be involved in MMVD progression. While they were originally discovered because of their capacity to mediate bone and cartilage formation, there is an increasing awareness of their role in non-osteogenic processes, such as heart development, circulation homeostasis and several cardiovascular diseases (120). Similar to TGF-βs BMPs ligands can bind to the heterotetrameric transmembrane receptor complexes comprised of two serine-threonine kinase type I and type II receptors, resulting in initiation of canonical or non-canonical signaling cascades, including the downstream phosphorylation of R-SMAD effector proteins and the three well-characterized ERK, JNK and p38 MAPK pathways (121–123). Following activation of R-SMADs, also known as SMADs 1, 5, and 8, BMPs form heteromeric complexes by binding with the co-Smad mediator, Smad4, and translocate into the nucleus to mediate transcription of BMP-responsive genes (Figure 3) (124).

The potential role of BMP signaling was first recognized in MMVD degeneration in a Fbn-1 mutant murine model of MFS. Several studies have shown that BMP 2, 4, and 6 can contribute to cardiac valve development and EndoMT involving a key contribution of Smad1/4/5-dependent BMP signaling (125–129). Although the molecular mechanisms in which the BMPs might impact MMVD pathogenesis have not been clarified some studies suggest that non-canonical BMP4/SOX9 signaling regulates the phenotypic change in VICs and ECM remodeling in human myxomatous mitral valve tissue, with increased gene and protein expression of BMP4, Sox9, CRTAC1, CTGF, α-SMA, vimentin and desmin (67). BMP4 treatment itself results in increased expression of Sox9 and other markers of ECM reorganization and VIC activation in human MMVD valves (67). BMP2 signaling in the human endocardial lineage is essential for remodeling of atrioventricular valves since BMP2 knockout mice show reduced Sox9 expression and mitral valve malformation deficiencies (130). BMP2 and TGF-β1 synergistically stimulate the expression of the transcriptional factors SOX9, Twist1, and Snail1/2 and initiate EndoMT *via* canonical Smad1/5- and Smad2/3-dependent pathways (101). TGF-β induces quiescent VIC activation by BMP2 stimulation in deformed mitral valves in Fstl1-deficient transgenic mouse model indicating a potential molecular target for myxomatous mitral valve disease (101). Comparable activity of canonical TGF-β and BMP signaling has been detected in surgically excised human MMVD tissue (84).

### Other Important Pathways in Myxomatous Mitral Valve Degeneration

Several important regulatory pathways besides TGF-β signaling have also been shown to participate in the development of heart valves and progression of MMVD under certain specific cellular contexts. These molecular pathways include Wnt/β-catenin and Notch pathways (42, 84, 131). A microarray pathway analyses showed that the expression level of Wnt ligand (Wnt9A) and its receptor (frizzled 8), accompanied by upregulation of the extracellular positive modulator R-Spondin 2 and the target gene runt-related transcription factor 2 (Runx2), were increased in human MMVD tissues. These observations were further supported by the increased expression of Wnt9A, β-catenin and Wnt-target gene WISP1 in TGF-β2-treated human mitral VICs and Ang II-induced myxomatous degeneration in murine mitral valves (84). The Notch pathway also plays an important role in the development of outflow tract of the heart which starts with EndoMT in the endocardial cells leading to the formation of cardiac valves (132). However, there lacks enough evidence that Notch signaling is directly involved in the onset and progression of MMVD in human and dogs. To what extent TGF-β signaling might interact with these pathways is beyond the scope of this review.

## Conclusion

MMVD is the most important acquired mitral valve disease in both dogs and humans and its natural history, pathogenesis and progression, pathology, cell and molecular changes are reasonably well characterized. Current areas of investigation are focused on genetic analysis, small and large animal models, the role of TGF-β dysregulation, abnormal EndoMT, and the impact of biomechanical strain in the pathogenesis of MMVD. There is also parallel interest in monocyte infiltration, particularly macrophages, as potential contributors to MMVD pathogenesis, at least in human mitral valvulopathies. While transgenic rodent models and surgically resected human valve tissues give insight into many molecular aspects of MMVD they are limited in modeling the chronicity of this disease and the extensive secondary fibrosis in patient-derived human valve tissues hampers examination of molecular events controlling myxomatous initiation and progression. Interestingly, much more is known about the earlier stages of the disease in the dog, and the naturally occurring disease in the dog might be the best large animal model to study human MMVD considering the shared cellular and molecular events in the two species. The important role of TGF-β signaling in the onset and progression of MMVD has been emphasized by studies using mouse models, valve samples of human and canine myxomatous mitral valves and in *in vitro* canine aVIC cell culture models. Phenotypic transition of qVICs to aVICs induced by dysregulated TGF-β signaling appears to be a key contributor to valve myxomatous degeneration though aberrant matrix remodeling, exerting control through complex canonical and non-canonical signaling pathways interactions and effects, which can conceivably affect the disease phenotype alone or in combinations. To what extent one of these might be a dominant pathway for the diseased is still unknown. Furthermore, what abnormal signaling contributes to the survival and persistence of aVICs in diseased valves remains unanswered. Understanding the mechanisms that control cell persistence in this disease likely will give clues to the pathogenesis and identify potential therapeutic targets in both the dog and human.

## Data Availability Statement

The original contributions presented in the study are included in the article/supplementary material, further inquiries can be directed to the corresponding author/s.

## Author Contributions

QT: major contribution to writing and preparation. AM, KP, and VEM: additional writing and editing. BMC: initiator of review, major contribution to writing and final editing. All authors contributed to the article and approved the submitted version.

## Conflict of Interest

The authors declare that the research was conducted in the absence of any commercial or financial relationships that could be construed as a potential conflict of interest.

## Publisher’s Note

All claims expressed in this article are solely those of the authors and do not necessarily represent those of their affiliated organizations, or those of the publisher, the editors and the reviewers. Any product that may be evaluated in this article, or claim that may be made by its manufacturer, is not guaranteed or endorsed by the publisher.
